# Characterization and Pharmacokinetic Evaluation of Oxaliplatin Long-Circulating Liposomes

**DOI:** 10.1155/2021/5949804

**Published:** 2021-04-20

**Authors:** Nihad Cheraga, Ammar Ouahab, Yan Shen, Ning-Ping Huang

**Affiliations:** ^1^State Key Laboratory of Bioelectronics, School of Biological Science and Medical Engineering, Southeast University, Nanjing 210096, China; ^2^Department of Pharmaceutics, China Pharmaceutical University, Nanjing 210009, China

## Abstract

The clinical efficacy of Oxaliplatin (L-OHP) is potentially limited by dose-dependent neurotoxicity and high partitioning to erythrocytes *in vivo*. Long-circulating liposomes could improve the pharmacokinetic profile of L-OHP and thus enhance its therapeutic efficacy and reduce its toxicity. The purpose of this study was to prepare L-OHP long-circulating liposomes (L-OHP PEG lip) by reverse-phase evaporation method (REV) and investigate their pharmacokinetic behavior based on total platinum in rat plasma using atomic absorption spectrometry (AAS). A simple and a sensitive AAS method was developed and validated to determine the total platinum originated from L-OHP liposomes in plasma. Furthermore, long-circulating liposomes were fully characterized *in vitro* and showed great stability when stored at 4°C for one month. The results showed that the total platinum in plasma of L-OHP long-circulating liposomes displayed a biexponential pharmacokinetic profile with five folds higher bioavailability and longer distribution half-life compared to L-OHP solution. Thus, long-circulating liposomes prolonged L-OHP circulation time and may present a potential candidate for its tumor delivery. Conclusively, the developed AAS method could serve as a reference to investigate the pharmacokinetic behavior of total platinum in biological matrices for other L-OHP delivery systems.

## 1. Introduction

Oxaliplatin (L-OHP) is a third generation of platinum-based anticancer agents with a wide spectrum of antitumor activity and lower toxicity compared to previous generations, cisplatin, and carboplatin [1]. Nevertheless, its clinical efficacy is potentially limited due to its high and irreversible binding to plasma proteins and erythrocytes leading to low plasma concentrations [2]. In addition, as references reported, L-OHP showed dose-limiting side effects such as peripheral sensory neuropathy, thrombocytopenia, and gastrointestinal toxicity [3, 4]. Therefore, a drug delivery system that could improve its therapeutic index and decrease its toxicity is highly desirable.

Liposomes are one of the first drug delivery systems to show increased delivery of platinum-based anticancer drugs to solid tumors by altering their biodistribution [5, 6]. Liposomes have repeatedly shown their ability not only to improve the therapeutic index of anticancer drugs, but also to reduce toxicity by minimizing drug uptake by healthy tissues. During the past decades, several liposomal formulations have been clinically approved [7]. Recently, Lipoplatin, a cisplatin liposomal formulation, has reached phase III clinical trial [8, 9]. Moreover, L-OHP undergoes a rapid biotransformation *in vivo* due to its strong electrophilicity, yielding reactive platinum metabolites that can irreversibly bind to plasma proteins, erythrocytes, or other low molecular weight compounds [10, 11]. Therefore, the encapsulation of L-OHP into liposomes could improve its pharmacokinetic profile and thus enhance its therapeutic efficacy [12]. Besides, the lipid bilayer would serve as a barrier preventing drug leakage in blood stream. Long-circulating liposomes or PEGylated liposomes can prolong the circulation time of drug in blood by preventing the rapid clearance of liposomes by the reticuloendothelial system (RES), leading to improved drug accumulation in the tumor interstitium by the enhanced permeability and retention (EPR) effect [13]. This phenomenon is mainly due to dysregulation of the vasculature in tumor tissues coupled with poor lymphatic drainage [14]. Recently, couple studies have reported the encapsulation of L-OHP into PEGylated liposomes, which showed significant tumor growth suppression *in vivo* when used in mono- or combination therapies [2, 15–17]. However, the pharmacokinetic profiles of these liposomes were not investigated [18]. In fact, understanding the pharmacokinetics of L-OHP long-circulating liposomes is necessary to provide scientific basis for future clinical applications [9].

It was reported that after intravenous infusion, L-OHP is mainly identifiable in three compartments, plasma-bound platinum, ultrafiltrate plasma platinum, and erythrocyte-bound platinum [19]. The ultrafiltrate platinum contains the intact drug and the low molecular weight platinum metabolites, which are generally known to be the main active ingredients, whereas platinum bound to plasma proteins or erythrocytes is considered to be pharmacologically inactive [10, 20]. Intact L-OHP was completely eliminated from blood circulation after 2 h of its intraperitoneal injection to rats [20]. Therefore, the quantification of only intact drug or only platinum metabolites projects a certain limitation on the real pharmacokinetic behavior of L-OHP *in vivo*. Hence, monitoring total platinum concentrations rather than intact parent drug (or a metabolite) is a generally accepted approach that has been adopted by several studies on the pharmacokinetics of platinum analogues [19, 21, 22]. Atomic absorption spectrometry (AAS) is the common used technique for specific determination of elemental platinum in biological matrices [23–25]. This technique is based on the light absorption of free platinum at a characteristic wavelength after atomization of biological samples [23]. To the best of our knowledge, there has been no attempt to investigate the pharmacokinetics of L-OHP long-circulating liposomes following intravenous bolus injection in rats using AAS method.

In this study, L-OHP PEGylated liposomes (herein referred as L-OHP PEG lip) and L-OHP nonPEGylated liposomes (herein referred as L-OHP bare lip) were prepared by reverse-phase evaporation method (REV). Subsequently, a full *in vitro* characterization study of both liposome formulations was conducted. Furthermore, we developed and validated a reproducible AAS method to trace elemental platinum in rat plasma. The validation was carried out according to the International Conference of Harmonization (ICH) guideline Q2(R1). Finally, we investigated the pharmacokinetic profile of L-OHP PEG lip based on total platinum in plasma and the best pharmacokinetic fit model.

## 2. Materials and Methods

### 2.1. Materials

Oxaliplatin (L-OHP) was purchased from Shandong Boyuan Chemical Co. (Shandong, China). Egg phosphatidylcholine (EPC, purity > 98%, PC 98T) and cholesterol (CHOL, 57-88-5, purity > 99%) were purchased from Shanghai Advanced Vehicle Technology (AVT) Pharmaceutical Ltd. (Shanghai, China). 1,2-Distearoyl-sn-glycero-3-phosphoethanolamine-N-[maleimide(polyethylene glycol)-2000] (DSPE-mPEG2000, purity > 99%, 147867-65-0) was purchased from Avanti Polar Lipids, Inc. (Alabama, USA). Fetal bovine serum (FBS) was purchased from Gibco (Grand Island, USA). Chloroplatinic acid (Platinum Standard containing 1000 *μ*g/mL) was obtained from nonferrous metals and electronic materials analysis and testing center (Nanjing, China). HNO_3_ and Triton X-100 were purchased from Nanjing Chemical Reagent (Nanjing, China). Glucose 5% for injection was purchased from Anshe Shuangji Pharmaceutical Ltd. (China). Ultrapure water to a resistivity of 18 M*Ω*.cm was used for all experiments (Milli-Q Plus System, Billerica, USA). All other reagents were of analytical grade and used without further purification.

### 2.2. Animals and Ethical Statement

12 Sprague Dawley (SD) rats weighting about 200 ± 20 g were purchased from Shanghai SIPPR-Bk Lab Animal Co., Ltd. (Shanghai, China). The authors declare that all the experimental procedures were approved by the Animal Welfare and Research Ethics Committee of Southeast University (No: 20170301006). All the animal experiments were conducted in full compliance with the ethical guidelines of Southeast University.

### 2.3. Preparation of Liposomes

L-OHP bare and L-OHP PEG liposomes were composed of EPC : CHOL (2 : 1 molar ratio) and EPC : CHOL : DSPE-mPEG2000 (2 : 1 : 0.2 molar ratio), respectively. All liposomes were prepared by reverse-phase evaporation method (REV) developed by Szoka and Papahadjopoulos with a little modification [26]. Briefly, lipids (300 mg) were dissolved in a mixture of chloroform/diethyl ether (1 : 3 v/v); then, 3 mL L-OHP solution (5 mg/mL) in 9% sucrose was dropped into the lipid mixture to form w/o emulsion. The volume ratio of aqueous to organic phase was maintained to 1 : 3. The emulsion was then sonicated for 5 min at 200 W (3 s on 2 s off) using a probe type sonicator (Nanjing Xianou instruments, China). Liposome suspensions were obtained by evaporation of the organic solvents using a rotary evaporator under reduced pressure at 40°C for 1 h. The resulting liposomes were extruded through 0, 22 *μ*m polycarbonate membrane. The nonencapsulated L-OHP was removed by dialysis (MWCO 14,000) against 1 L of 9% sucrose for 2 h under constant stirring with renewing the dialysis solution every 30 min.

### 2.4. Characterization of L-OHP Liposomes

#### 2.4.1. Particle Size and Zeta Potential

Particle size and polydispersity index (PDI) were determined by dynamic light scattering (DLS) using particle analyzer (Brookhaven Instruments, USA) at 25°C and scattering observed at 90° angle with respect to the incident beam. L-OHP liposome preparations were diluted 100 times with Milli-Q water prior measurement. Zeta potential was measured using a ZetaPlus *ζ* potential analyzer (Brookhaven Instruments, USA) at 25°C. All measurements were performed in triplicate, and results were reported as mean ± SD.

#### 2.4.2. Encapsulation Efficiency and Drug Loading

The encapsulation efficiency was determined by modified ultrafiltration centrifugation method [27]. In brief, 200 *μ*L of initial L-OHP liposomes without dialysis was diluted 10 folds with 9% sucrose, then centrifuged at speed of 12000 rpm for 10 min at 4°C using Amicon® ultra-2 filter devices (Merck Millipore, 10,000 MWCO). The filtrate was collected and used to assess free L-OHP concentration. To evaluate the efficiency of this method, we quantified the recovery of L-OHP from free drug solutions and from the mixture of blank liposomes with free L-OHP (1 mg/mL) using the Amicon® devices. Another 200 *μ*L from L-OHP-liposomal suspensions without dialysis was disturbed by 10% Triton X-100 at 60°C for 10 min to ensure the complete release of encapsulated L-OHP. After cooling at room temperature, the solution was diluted and the concentration of this solution was used to measure total L-OHP concentration. Free and total L-OHP were analyzed using a validated HPLC method (supplementary materials). The separation was performed in inertsil ODS C18 column (4.6 mm × 250 mm, 5 *μ*m). The mobile phase was a mixture of water and methanol (95 : 5, v/v) at a flow rate of 1 mL/min. The detection wavelength was 250 nm, and the injection volume was 20 *μ*L. *C*_free_ and *C*_tot_ are the concentration of nonencapsulated L-OHP and the total L-OHP concentration in the liposomal suspension without dialysis, respectively. The percentage of encapsulation efficiency (EE) was calculated as follows:

(1) $$\begin{array}{c} \text{EE }\left(\%\right)=\frac{C_{\text{free}}-C_{\text{tot}}}{C_{\text{tot}}}\times 100. \end{array}$$

The drug loading (DL) was calculated according to the below equation:

(2) $$\begin{array}{c} \text{DL}\left(\%\right)=\frac{W_{\text{free}}-W_{\text{tot}}}{W_{\text{lip}}}\times 100, \end{array}$$

where *W*_free_ and *W*_tot_ are the amount of nonencapsulated and total L-OHP, respectively, and *W*_lip_ is the weight of lyophilized liposomes.

#### 2.4.3. Transmission Electron Microscopy (TEM)

Liposomes were observed by transmission electron microscopy (TEM). L-OHP liposomes were diluted with Milli-Q water, dropped on a carbon-coated copper grid, and air-dried at room temperature. The negative contrast staining was carried out using 2% aqueous phosphotungstic acid solution, followed by air direness and examination using TEM (Hitachi-7650, Japan) operating at 80 kV.

#### 2.4.4. Differential Scanning Calorimetric (DSC)

The thermal behavior of the blank liposomes, L-OHP bare lip, and L-OHP PEG lip was evaluated using a differential scanning calorimeter (204 phoenix, NETZSCH Scientific Instruments, USA). 3 mg of the samples was placed in an aluminum pan along with the standard reference aluminum, and the DSC was recorded between 0°C and 350°C at a scan rate of 10°C/min for three cycles.

### 2.5. *In Vitro* Release

Due to the hydrolysis of L-OHP in PBS medium, 9% sucrose solution, a nonionic medium, was selected to conduct the *in vitro* release. Briefly, 1 mL of liposome dispersion was placed into a dialysis membrane bag (MWCO 14,000) [28]. Then, the end-sealed bags were immersed in 50 mL of release medium and kept at 37°C under constant shaking in a water bath at 100 rpm. 1 mL sample was withdrawn at predetermined time intervals and replaced with an equal volume of fresh medium. The amount of L-OHP released from liposomes was determined by HPLC method described above. The cumulative release was built as the percentage of release at each sampling time applying the following formula [17]:

(3) $$\begin{array}{c} \text{The cumulative release }\left(\%\right)=\frac{W_{t}}{W_{i}}, \end{array}$$

where *W*_*t*_ is the cumulative amount of L-OHP released at time *t* and *W*_*i*_ is the total amount of L-OHP initially loaded into the dialysis bag. All the experiments were performed in triplicate.

### 2.6. Stability Study

The influence of serum proteins in the stability of L-OHP liposomes was evaluated in 100% FBS (fetal bovine serum) [29]. 200 *μ*L of the liposomal formulations was added to 2 mL of 100% FBS solution and incubated at 37°C with constant stirring for 4 h. At indicated time points, 200 *μ*L of the suspension was diluted 50 times with Milli-Q water and the particle sizes and PDIs were measured as the evaluation criteria.

The physical stability was also evaluated upon storage of both liposomes in air-tight sealed vials at 4°C and at 25°C for one month. The assessment was based on the visual inspection, particle size, and drug retention.

For chemical stability, the extent of lipid peroxide value (POV) was monitored by measuring the absorbance of thiobarbituric acid reactive species (TBARS) formed by UV spectrometry, according to modified procedure [30, 31]. TTH reagent was prepared by dissolving 30 mg of trichlorometric acid (TCA) and 750 mg of TBA (thiobarbituric acid) in 0.25 N HCL solution at 50°C, and the volume was completed to 200 mL. Then, 5 mL of this reagent was added to 1 mL of the prepared liposomes. The mixture was incubated at 100°C for 30 min and allowed to cool at room temperature. The TTH solution was used to complete the volume to 10 mL. Then, tubes were centrifuged at 4100 rpm for 5 min, and the supernatant was assessed at 535 nm. The absorbance was recorded as PVO; TTH solution was used as blank.

### 2.7. Osmolality of L-OHP Liposomes

The osmotic pressure of L-OHP liposomes was determined using freezing point depression STY-1 osmometer (Tianda technology, China). The instrument was precalibrated with standard solutions (100, 200, and 300 mOsm/kg). Three different preparations of L-OHP bare lip and L-OHP PEG lip were analyzed.

### 2.8. Pharmacokinetic Study

#### 2.8.1. Sample Processing

SD rats were randomly divided into three groups (*n* = 4) and received an intravenous (IV) bolus injection via teal vein of either L-OHP solution, L-OHP bare lip, or L-OHP PEG lip at L-OHP dose of 10 mg/kg (equivalent to a platinum dose of 4.9 mg/kg). Blood samples were collected from orbital sinus using heparinized capillary tubes at 5, 15, 30, and 60 min and 2, 4, 6, 8, 10, and 24 h after drug administration. Plasma was obtained by immediate centrifugation at 3500 rpm for 5 min at 4°C. All samples were stored at -20°C until analysis.

To reduce matrix interference and minimize platinum loss during charring/atomization, Triton X-100 was used as a matrix modifying. 0.5% Triton X-100 aqueous solution mixed with plasma at 1 : 1 volume ratio was defied as the optimal condition to reduce background signal. In brief, 200 *μ*L plasma was mixed with 200 *μ*L 0.5% Triton X-100 solution; then, the volume was completed to 10 mL with 5% nitric acid solution (corresponding to 50-fold dilution). When the platinum concentration was expected to be below LOQ, the plasma samples were diluted 10 folds. After the acid digestion, mixtures were vortex-mixed for 30 s and aliquots were analyzed by AAS for platinum content.

#### 2.8.2. Stock and Working Solution

Instead of L-OHP, Platinum Standard (chloroplatinic acid 1000 mg/L) was used as a standard for the preparation of platinum calibration standards in plasma and quality-control (QC) samples. A stock solution of 10 *μ*g/mL was prepared using 5% nitric acid solution. The solution was further diluted to obtain a working solution at 1 *μ*g/mL platinum concentration. Platinum stock solution and working solution were stored at 4°C. From the working solution, 0.1, 0.2, 0.4, 0.6, 0.8, 1, and 1.2 mL were placed in 10 mL volumetric flasks. Then, 200 *μ*L of blank plasma and 200 *μ*L of matrix modifier solution were added in each volumetric flask. After vortex-mixing for 30 s, the volume was completed to the mark with 5% nitric acid to obtain platinum calibration standards in blank plasma ranging from 10 to 120 ng/mL (corresponding to 500-6000 ng/mL in undiluted matrix).

#### 2.8.3. Instrumentation and Operating Conditions

Total platinum concentration in rat plasma was measured with Thermo Electron iCE 3300 Graphite Furnace-atomic absorption spectrophotometer GF-AAS (Thermo Electron iCE 3300, Cambridge, UK) equipped with a GFS33 integrated autosampler. Pyrolytically coated graphite tubes (Thermo Electron) were used. The measurements were carried out with a platinum hollow cathode lamp operated at 12 mA and at a wavelength of 265.9 nm with a monochromator slit width seat at 0.7 nm. The temperature program of the instrument comprised drying, ashing, atomization, and cleaning-cooling stages (Table 1). During the atomization stage absorbance was monitored using Deuterium background correction. The inert carrier gas argon was used to purge the graphite tube at a flow rate of 0.2 L/min. The gas flow was turned off during the atomization stage. A total volume of 20 *μ*L consisted of mixture of matrix modifier solution, plasma sample, and diluent (5% nitric acid) was introduced into the graphite tube by the autosampler. Data were acquired using the SOLAAR House software version 2.01 (Cambridge, UK) and processed using the SOLAAR Data Station version 10.02 software (Cambridge, UK).

#### 2.8.4. Method Validation

The validation of the analytical procedure was carried out in plasma according to the International Conference of Harmonization (ICH) guideline Q2(R1) [32].

*(1) Sensitivity*. The limit of detection (LOD) was determined as the concentration with a signal-to-noise ratio of 3, while the limit of quantification (LOQ) was determined as the concentration with a signal-to-noise ratio of at least 10. The accuracy should be 20% of the actual value with a precision not exceeding 20%.

*(2) Linearity*. The linearity test was carried out with seven sets of calibration standards at concentration ranging from 10 to 120 ng/mL, which were analyzed in three different runs. The calibration curve was evaluated by linear regression of the plot of platinum calibration standards' concentrations (*x*) versus the heights of the corresponding absorbance peaks (*y*).

*(3) Precision and Accuracy*. Accuracy and precision (within-run and between-run) of method were determined by the analysis of QC samples (LOQ, medium and high). The platinum working solution was used to spike plasma to obtain QC samples at three concentrations (10, 60, and 100 ng/mL). Six replicates of each concentration were processed and analyzed as described above. Then, each concentration was analyzed in six different days. Accuracy was determined as the percentage of the nominal concentration. Accuracy should be within 80–120% of the nominal concentration for the LOQ and within 85–115% of the nominal concentrations for other concentrations. Precision was expressed as the relative standard deviation (RSD) and should not exceed 20% for the LOQ and 15% for the other concentrations.

*(4) Specificity*. To investigate whether endogenous matrix constituents interfered with the assay, blank plasma samples and blank plasma samples spiked with L-OHP at the LOQ level were analyzed. The peak heights for the blank matrix should not exceed 20% of peak height at the LOQ level.

*(5) Recovery*. The recoveries of the acid extraction of platinum from plasma were determined by comparing the concentration of extracted samples (spiked before extraction) to that of unextracted samples (spiked after extraction) at three QC levels (10, 60, and 100 ng/mL).

#### 2.8.5. Pharmacokinetic Parameters and Statistical Analysis

Pharmacokinetic parameters were calculated by the PK Solver 2.0 add-in of MS-Excel (Nanjing, China) using compartmental analysis. Herein, the best pharmacokinetic model fitting plasma concentration data was selected based on Schwarz criterion (SC) and Akaike's information criterion (AIC). The GraphPad Prism 6.0 Software (San Diego, USA) was used for statistical analysis. Student's *t*-test and one-way ANOVA were used to analyze comparisons between two groups and calculate differences among groups, respectively. Statistical significance was set at *P* < 0.05. Data are expressed as mean ± SD.

## 3. Results and Discussion

### 3.1. Characteristics of L-OHP Liposomes

**Figure 1:**
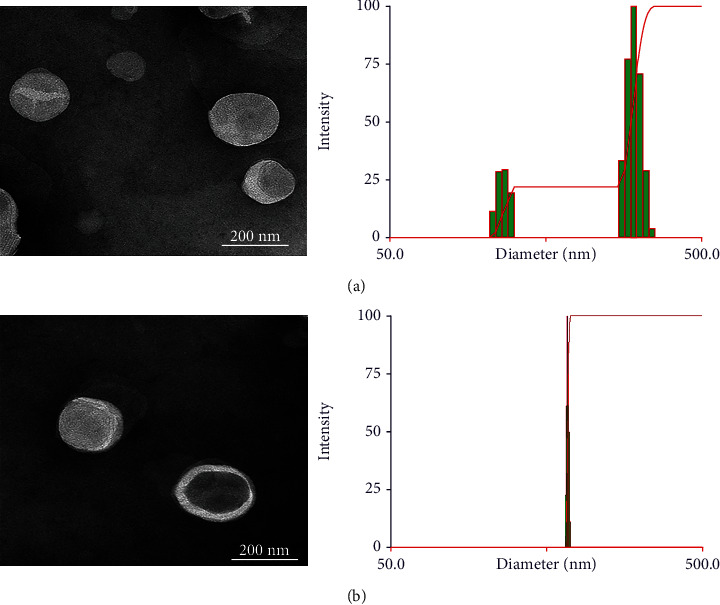
Characterization of L-OHP liposomes. TEM image and size distribution of L-OHP bare lip by DLS (a). TEM image and size distribution of L-OHP PEG lip by DLS (b).

The physicochemical characteristics of both L-OHP liposomes are summarized in Table 2. Compared to bare liposomes, the size distribution by intensity of L-OHP PEG lip showed a unimodal distribution with a low polydispersity suggesting better homogeneity of the liposomal suspension (Figure 1). This could increase the liposome stability and decrease the aggregation of vesicles. The mean particle size obtained from DLS analysis was 235 ± 20.30 and 204 ± 1.10 for L-OHP bare lip and L-OHP PEG lip, respectively. A slight decrease in the particle size was observed following the inclusion of DSPE-mPEG2000 to liposomal membrane. This may be explained by the steric repulsion among PEG chains exposed from the outer leaflet of the liposomal membrane [33]. The zeta potential of L-OHP bare liposomes was about -8.4 mV, which was expected due to the low negative charge of EPC. The decrease in the zeta potential upon PEGylation could be ascribed to the negatively charged phosphate group of DSPE-mPEG2000, which was in accordance with the result reported in literature [34]. The EE of L-OHP in bare liposomes was 25.40%, 26.20% in PEGylated ones, indicating that the inclusion of PEG had no significant influence on drug encapsulation. The DL was 0.92% and 0.98% for L-OHP bare lip and L-OHP PEG lip, respectively. The morphology of the different liposomal preparations was observed by TEM, and the images showed regular spherical shapes with large internal space (Figure 1). However, a white film surrounding L-OHP PEG lip was observed, which could be attributed to PEG chains coating the surface. The size determined from TEM images using the Image J software was smaller than the one by DLS (Table 2), and this could be explained by the shrinkage of the liposomes during the drying process [35].

### 3.2. Differential Scanning Calorimetric (DSC)

**Figure 2:**
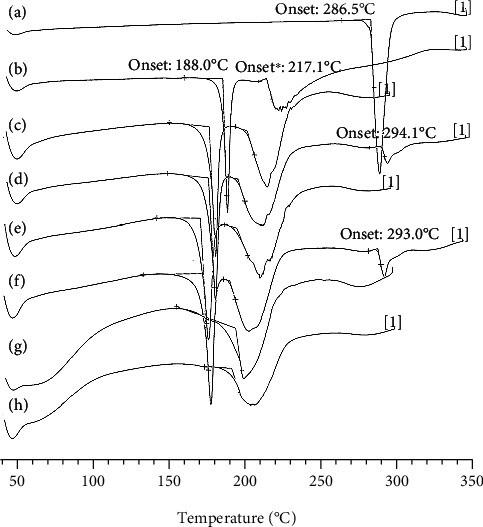
Differential scanning calorimetry (DSC) thermograms of (a) L-OHP, (b) sucrose, (c) blank bare liposomes, (d) the physical mixture of blank bare liposomes and L-OHP, (e) blank PEG liposomes, (f) the physical mixture of blank PEG liposomes and L-OHP, (g) L-OHP bare liposomes, and (h) L-OHP PEG liposomes.

As presented in Figure 2, the thermogram of L-OHP displayed an endothermic peak at 286.5°C corresponding to its melting point. Sucrose had displayed two peaks at 188.0°C and 217.1°C. Blanks bare and PEGylated liposomes showed two endothermic peaks at 179.4°C and 204.9°C and at 173.3°C and 197.7°C, respectively. Consequently, the physical mixture of blank liposomes with L-OHP displayed three peaks with a little modification of the melting temperatures probably due to drug/phospholipid interaction. Interestingly, the thermal profiles of both L-OHP liposomes exhibited a single endothermic peak, and no peak representing the melting of the drug was observed. The endothermic peaks of L-OHP bare lip and L-OHP PEG lip were both shifted to lower temperatures. These results suggest that L-OHP interacts with lipid phase when it is encapsulated into the aqueous phase of liposomes. Moreover, the interaction of L-OHP with phospholipids adding to the presence of sucrose in the preparation may have decreased the liposomal decomposition temperature. In fact, the DSC analysis shows that L-OHP was encapsulated into liposome core and thereby protected from temperature decomposition.

### 3.3. *In Vitro* Release

**Figure 3:**
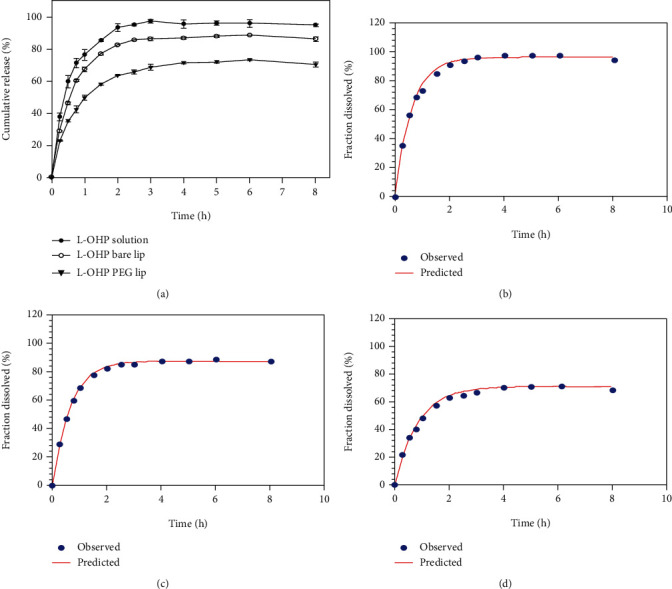
*In vitro* drug release profile of L-OHP, L-OHP bare liposomes, and L-OHP PEG liposomes in 9% sucrose solution at 37°C (a). First-order model fitting curves of *in vitro* cumulative release data of (b) L-OHP solution, (c) L-OHP bare liposomes, and (d) L-OHP PEG liposomes.

The *in vitro* cumulative release profiles of L-OHP from different formulations in 9% sucrose displayed biphasic patterns and are shown in Figure 3(a). Free L-OHP was completely released after 2 h, whereas 80% and 62% of total L-OHP were released from bare and PEGylated liposomes, respectively, during the same time period. Zhang et al. reported that L-OHP solution was completely released within 4 h, while L-OHP-loaded liposomes released nearly 60% of the drug within 0.5 h followed by complete release within 24 h [28]. These liposomes showed higher burst release than our reported L-OHP bare lip where nearly 30% of L-OHP was released within 0.5 h (Figure 3(a)). This difference could be explained by a possible incomplete removal of nonencapsulated L-OHP from the formulation, as well as the difference in the EPC : CHOL lipid ratio used for liposome preparation. Our L-OHP bare lip had higher cholesterol content, which can decrease the fluidity and the permeability of phospholipid membranes to small molecules and hydrophilic drugs [36]. Furthermore, the hydrophilic nature of L-OHP could be considered as one of the causes for its fast release [37]. It was reported that water soluble molecules diffuse rapidly through liposome bilayers [38]. A steady release followed the initial burst with a release rate attending 86% and 70% for L-OHP bare lip and L-OHP PEG lip, respectively. The release rate of L-OHP from bare liposomes was faster than that from PEGylated ones. This was attributed to liposomal membrane composition, where the inclusion of DSPE-mPEG2000 could result in a higher membrane rigidity and decreased permeability leading to a slower release behavior. The release mechanism was further investigated by fitting the release data to first order Korsmeyer-Peppas and Weibull models using the DD Solver software (Nanjing, China) (Table 3). According to *R*^2^ closer to 1, all three formulations best fitted the first-order model (Figures 3(b)–3(d)), followed by Weibull and Korsmeyer-Peppas models. The drug release mechanism was evaluated by the exponent parameter (*β*) in Weibull model equation [39]. The exponent was between 0.39 and 0.69 suggesting that L-OHP release from bare and PEGylated liposomes was governed by diffusion mechanism.

### 3.4. Stability

#### 3.4.1. Short Storage

**Figure 4:**
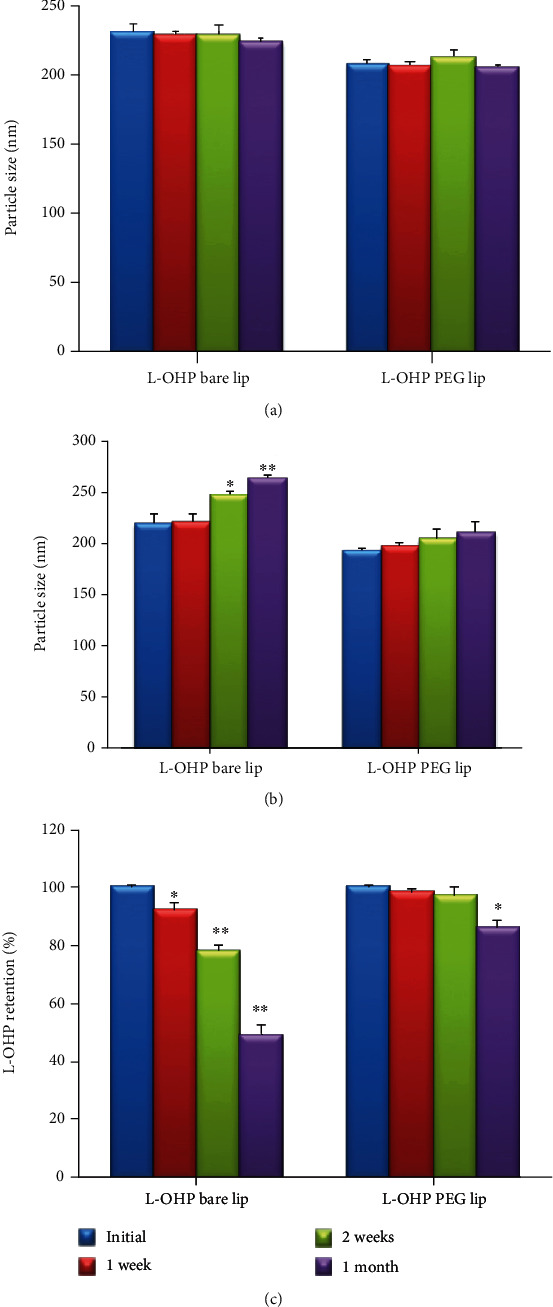
(a) Particle size of L-OHP liposomes stored at 4°C for one month. (b) Particle size of L-OHP liposomes stored at 25°C for one month. (c) Retention of L-OHP after one month storage at 4°C. Data is shown as mean ± SD (*n* = 3). ^∗^Statistically significant difference compared with initial week (^∗^*P* < 0.05 and ^∗∗^*P* < 0.01).

The typical phenomenon of liposome instability is the aggregation or fusion of vesicles during storage. On the first visual inspection, L-OHP bare lip appeared as translucent yellow dispersion, while L-OHP PEG lip appeared as milky transparent dispersion. No aggregations or precipitations were observed when both liposomes were stored at 4°C for one month. However, aggregations were observed when L-OHP bare lip was stored at 25°C. Moreover, no significant change was observed in the size of both liposomes when stored at 4°C for one month (Figure 4(a)). However, in contrast to PEGylated liposomes, the size of L-OHP bare lip significantly increased when stored at 25°C (Figure 4(b)). This could be explained by the PEG steric barrier and the negative zeta potential leading to electrostatic repulsion between particles [33, 38]. On the other hand, the stability of liposomes was evaluated in terms of drug retention (Figure 4(c)). L-OHP leaked from bare liposomes with 49.65% of drug remaining after one month storage at 4°C. In the case of PEGylated liposomes, 86.32% of L-OHP remained after one month storage. Interestingly, this leakage was not accompanied with changes in particle size at 4°C (Figure 4(a)), and hence, it was assumed that L-OHP leakage from PEGylated liposomes was not due to the liposome aggregation, but rather to its diffusion cross the membrane.

#### 3.4.2. In Fetal Bovine Serum (FBS)

**Figure 5:**
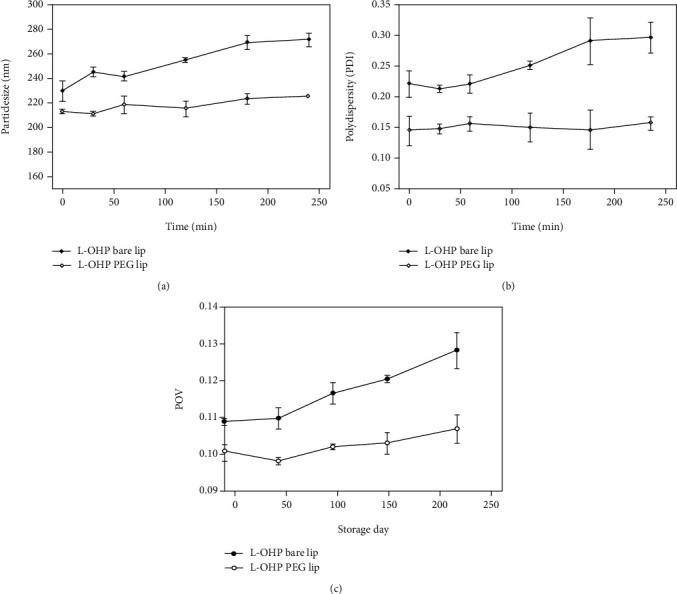
(a) Particle size of the L-OHP liposomes after incubation at 37°C in serum. (b) Polydispersity changes of L-OHP liposomes after incubation at 37°C in serum. (c) The peroxide value (POV) change of L-OHP liposomes after storage at 4°C for one month. Data is shown as mean ± SD (*n* = 3).

Blood is a complex mixture of molecules, minerals, vitamins, and proteins, which can affect the stability of the liposomes in blood stream. The changes in size and PDI of liposomes when incubated in FBS for 4 h are shown in Figures 5(a) and 5(b), respectively. The size of L-OHP bare lip increased from 230 nm to 271 nm with an obvious increase in the PDI, whereas the size of PEGylated liposomes showed no significant change. It was reported that protein adsorption would mask or neutralize the surface charge leading to liposome aggregation and thereby resulting in size increase [40]. However, this phenomenon is limited when the surface is stealthed with PEG hydrophilic chains, which would delay protein adsorption and thus increase the colloidal stability in serum [38].

#### 3.4.3. Liposome Peroxidation

In this study, the oxidation of lipids was monitored by assessing the peroxide value (POV) of both L-OHP liposomes stored at 4°C every week for one month (Figure 5(c)). L-OHP bare lip showed a significant increase of POV value from 0.108 to 0.128, whereas L-OHP PEG lip showed a reduced lipid peroxidation when stored at the same condition as bare liposomes. This could be explained by the difference in the lipid composition of the liposomal membranes. In the case of PEGylated liposomes, it is less likely for DSPE, as a synthetic saturated phospholipid, to form peroxidation products upon exposure to oxygen [41].

### 3.5. Osmolality of L-OHP Liposomes

As illustrated in Table 4, the osmolality of L-OHP bare lip was about 320 ± 1.52 mOsm/kg, while the one of L-OHP PEG lip was 338 ± 4.72 mOsm/kg. The human plasma osmolality is about 300 mOsm/kg [42]. When a solution is isotonic, it will diffuse out of the blood vessels after intravenous injection in similar way to the component of blood serum itself (ex., 9% NaCl solution). According to the Infusion Nursing Society (INS) standards, infusion and injection with an osmolality greater than 600 mOsm/kg will be responsible of vascular complications especially peripheral phlebitis [43]. In humans, the lowest risk of phlebitis occurred with solution of <450 mOsm/kg [44]. The osmolality of both L-OHP liposomes was within the recommendation limits, and thus, they are safe for intravenous injection.

### 3.6. Validation of AAS Method

#### 3.6.1. Sensitivity

The LOD of platinum in rat plasma was 2.38 ng/mL. The LOQ of the method was set at a platinum concentration of 10 ng/mL determined with a signal-to- noise ratio of 10. LOQ was chosen as the lowest concentration of the standard calibration curve in plasma.

#### 3.6.2. Linearity

The platinum calibration curve in plasma showed a good linearity in the dynamic range 10-120 ng/mL (corresponding to 500-6000 ng/mL in undiluted matrix), with a correlation coefficient of *R*^2^ = 0.9993 (Figure S4). The regression equation was *y* = 0.0018*x* + 0.0012. Samples having concentrations higher than the upper limit of linearity were appropriately diluted before the analysis, to avoid saturation of the signal intensity and unacceptable memory effect observed in subsequent atomization.

#### 3.6.3. Accuracy and Precision

Intra-assay and interassay precision and accuracy are summarized in Table 5. Overall, the intra-assay accuracy and precision was in the range 91.79-107.58% and 2.35-4.31%, respectively. The corresponding values for the interassay gave an accuracy of 99.01-104.81% and precision of 1.85-9.81%. The observed values for all QC levels were within the acceptance criteria for bioanalytical method validation [32].

#### 3.6.4. Specificity

The specificity of the AAS method was demonstrated by the lack of platinum specific absorbance in blank plasma samples (Figure S5 and S6), revealing no interference from endogenous material in the plasma matrix at the absorbance wavelength of platinum (265.9 nm).

#### 3.6.5. Recovery

The extraction recoveries of platinum from rat plasma matrix are listed in Table 6. The recovery should be in the range of 80-120% for LOQ and within 85-115% for the other concentration levels [32]. The results showed that acid digestion recoveries are within the aforementioned range.

In conclusion, these results suggest that the present AAS method is sensitive, accurate, and reproducible for quantifying the platinum originating from L-OHP in rat plasma.

### 3.7. Pharmacokinetics

**Figure 6:**
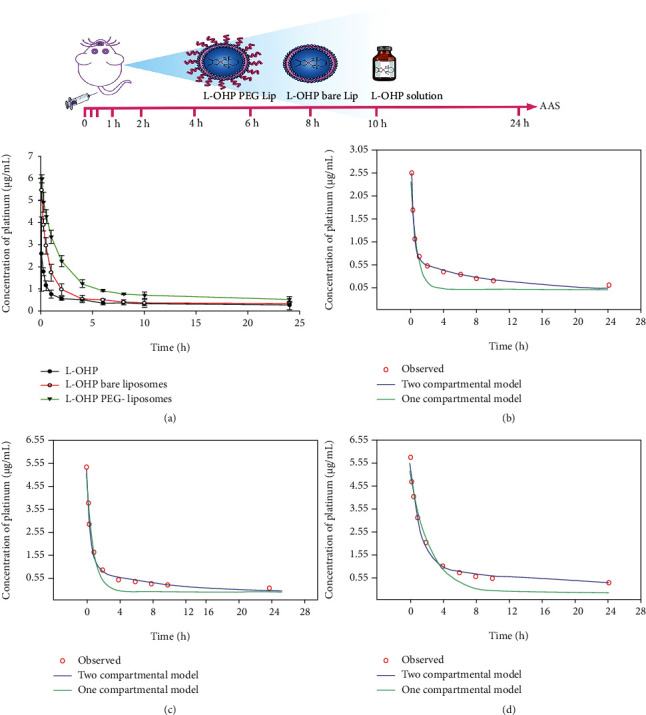
(a) Total platinum concentration-time curves of L-OHP, L-OHP bare liposomes, and L-OHP PEG liposomes in plasma following intravenous administration to SD rats at L-OHP dose of 10 mg/kg. Data is shown as mean ± SD (*n* = 4). Compartmental models fitting curves of the pharmacokinetic data of (b) free L-OHP solution, (c) L-OHP bare liposomes and (d) L-OHP PEG liposomes.

Total platinum concentration-time profiles of L-OHP, L-OHP bare lip, and L-OHP PEG lip in plasma after an IV injection to rats are presented in Figure 6(a). The total platinum concentrations decreased rapidly within the first 2 h and then declined slowly with a long terminal phase. AIC and SC were used as parameters to select the best-fit model for the pharmacokinetic data [45]. The smaller the AIC value is, the better the model is fit [45]. As illustrated in Table 7, the pharmacokinetic behavior of total platinum in plasma from free L-OHP, L-OHP bare lip, and L-OHP PEG lip compiled with the two-compartment model, characterized by a biphasic exponential decay with an initial rapid phase followed by a slower elimination terminal phase (Figures 6(b)–6(d)). The prolonged terminal elimination phase presumably represents inactive platinum complexes that were formed through chemical reactions with low molecular weight nucleophiles [20].

The pharmacokinetic parameters of platinum in plasma for free L-OHP, L-OHP bare lip, and L-OHP PEG lip were determined by compartmental method, and the results are listed in Table 8. The initial concentration (*C*_0_) of total platinum for free L-OHP (3.13 *μ*g/mL) was lower than that for L-OHP bare lip (5.99 *μ*g/mL) and L-OHP PEG lip (6.04 *μ*g/mL). This might be attributed to the extensive and irreversible uptake of free L-OHP by erythrocytes immediately after intravenous injection. As a matter of fact, a relatively low efficacy has been reported for L-OHP injection due to these pharmacokinetic limitations [46]. The encapsulation of L-OHP into bare and PEGylated liposomes in this work has effectively protected L-OHP in blood and achieved higher initial platinum concentrations. Nevertheless, when compared to bare liposomes, L-OHP PEG lip maintained significantly higher total platinum concentration in plasma throughout the study period (Figure 6(a)). These PEGylated liposomes increased the total platinum exposure in plasma with an AUC 5.1-fold higher than that of free L-OHP, whereas bare liposomes increased the AUC by 1.8-fold. Moreover, the distribution half-life (*t*_1/2_ *α*) of total platinum for L-OHP PEG lip (0.89 h) was longer than that for L-OHP bare lip (0.35 h) and free L-OHP (0.15 h), suggesting that PEGylated liposomes had a prolong circulation time in the blood probably due to the inclusion of DSPE-mPEG2000 into liposomal membrane. Similar observations of PEGylated liposomes extending platinum exposure and prolonging the circulation half-life *in vivo* of other platinum-based drugs were also reported [47, 48]. The presence of PEG, a hydrophilic polymer, provides a steric barrier at the liposome surface which reduces their clearance by the RES and thus ensures longer plasma half-lives [49]. Studies with PEG of different molecular weight from 120 to 5000 Da showed that the incorporation of PEG2000 into liposomes gave the highest drug blood levels [49]. Furthermore, our results have confirmed that PEGylated liposomes were eliminated rather slowly by an almost 8.4- and 2.5-fold lower clearance rate (Cl) as compared with free L-OHP and L-OHP bare lip, respectively. The enhanced long-circulation in the plasma could increase the L-OHP accumulation in the tumor area through EPR effect [46]. The pharmacokinetics of total platinum in this study are in accordance with the high L-OHP tumor accumulation, which correlated with an efficient antitumor activity *in vivo* of L-OHP-loaded PEGylated liposomal formulations, reported by other groups [2, 28, 46]. For instance, Zalba et al. found that the levels of L-OHP in the tumor at the end of the *in vivo* efficiency study were three times higher for PEGylated liposomes in comparison with free drug [2]. In another study, the amount of L-OHP in tumors was increased significantly when L-OHP was encapsulated into thermosensitive long-circulating liposomes [46]. Therefore, the pharmacokinetic evaluation with quantification of total platinum presented in this paper can support the findings of previous reports about of L-OHP long-circulating liposomes and help understand their efficacy and toxicity comprehensively. This work also provides an important reference for the pharmacokinetic study of other novel L-OHP delivery systems.

## 4. Conclusion

In this study, L-OHP was encapsulated into PEGylated and bare liposomes. Both liposomes were characterized in terms of particle size, zeta potential, morphology, *in vitro* release, osmolality, and chemical and physical stability. In addition, an accurate, reproducible, and sensitive AAS method for quantification of total platinum originated from L-OHP in rat plasma was developed and validated. The total platinum concentration-time curves for free L-OHP, L-OHP PEGylated, and bare liposomes all fitted with two-compartment model. L-OHP long-circulating liposomes significantly prolonged circulation time of total platinum in plasma with an AUC 5.1-fold higher than that of free L-OHP. In summary, L-OHP long-circulating liposomes showed longer circulation time, which could enhance liposome accumulation at the tumor area and thus improve L-OHP therapeutic index. Besides, this work also provides an important reference for the pharmacokinetic study of other novel L-OHP delivery systems.

## Figures and Tables

**Table 1:** Temperature program for AAS analysis of L-OHP in rat plasma.

Steps	Temperature (°C)	Time (s)	Ramp (°C/s)	Argon flow (L/min)	Read on
Drying 1	100	20	2	0.2	-
Drying 2	120	30	10	0.2	-
Charring	1400	30	150	0.2	-
Atomization	2700	3	0	0	Yes
Cleaning	2800	3	0	0.2	-

**Table 2:** Physicochemical characteristics of L-OHP liposomes (*n* = 3).

Formulation	Particle size (nm) by DLS	Particle size (nm) by TEM	PDI	Zeta potential (mV)	EE%	DL%
L-OHP bare lip	235.70 ± 20.30	109.05 ± 22.59	0.28 ± 0.01	−8.40 ± 1.52	25.40 ± 2.60	0.92 ± 0.14
L-OHP PEG lip	204.30 ± 1.10	112.89 ± 16.17	0.12 ± 0.05	−35.05 ± 2.26	26.20 ± 2.82	0.98 ± 0.10

DLS: dynamic light scattering; TEM: transmission electron microscopy; PDI: polydispersity index; EE: entrapment efficiency; DL: drug-loading.

**Table 3:** Fitting parameters of various kinetic models.

Formulation	First-order model		Korsmeyer-Peppas model			Weibull model	
	*R* ^2^ ^a^	*K* ^b^	*R* ^2^	*K*_KP_^c^	*n* ^d^	*R* ^2^	*β* ^e^
L-OHP	0.9947	1.847	0.9112	72.828	0.186	0.9961	0.580
L-OHP bare lip	0.9993	1.521	0.9109	62.768	0.219	0.9856	0.406
L-OHP PEG lip	0.9902	1.222	0.9377	47.908	0.258	0.9836	0.348

^a^Coefficient of correlation; ^b^first-order constant; ^c^release constant of Korsmeyer-Peppas model; ^d^diffusional exponent; ^e^exponent parameter in Weibull model.

**Table 4:** Osmolality of L-OHP liposomes.

Liposomes	L-OHP bare lip			L-OHP PEG lip		
Batch No.	1	2	3	1	2	3
Osmolarity (mOsm/kg)	322	321	319	344	335	337

**Table 5:** Precision and accuracy for quantification of platinum in rat plasma.

Nominal Conc. (ng/mL)	Conc. observed (ng/mL)	Precision (RSD %)	Accuracy (%)
Intra-assay (*n* = 6)			
10	9.18	2.28	91.79
60	64.55	4.01	107.58
100	104.50	4.31	104.50
Interassay (*n* = 6)			
10	9.9	9.81	99.01
60	60.07	1.85	100.12
100	104.81	3.79	104.81

**Table 6:** Extraction recoveries of platinum in rat plasma (*n* = 3).

QCs (ng/mL)	Recovery (%)	RSD (%)
10 ng/mL	95.53 ± 1.57	1.64
60 ng/mL	92.36 ± 2.9	3.14
100 ng/mL	91.49 ± 2.55	2.79

QC: quality-control.

**Table 7:** Akaike information criterion (AIC) and Schwarz criterion (SC) for each model.

Model	One compartment			Two compartments		
	AIC	SC	*R* ^2^	AIC	SC	*R* ^2^
L-OHP	-0.54	0.06	0.9476	-40.72	-39.51	0.9993
L-OHP bare lip	5.23	5.84	0.9603	-13.42	-12.21	0.9975
L-OHP PEG lip	9.33	9.94	0.9824	-8.61	-7.39	0.9980

AIC: Akaike information criterion; SC: Schwarz criterion.

**Table 8:** Pharmacokinetic parameters of total platinum in plasma after intravenous injection of L-OHP, L-OHP bare lip, and L-OHP PEG lip to rats (10 mg/kg, *n* = 4).

Parameters	L-OHP	L-OHP bare lip	L-OHP PEG-lip
*C*_0_ (*μ*g/mL)	3.135 ± 2.161	6.046 ± 0.871	5.994 ± 0.262
*t*_1/2_ *α* (h)	0.154 ± 0.071	0.347 ± 0.081	0.893 ± 0.100^∗∗∗^
*t*_1/2_ *β* (h)	5.899 ± 1.902	16.127 ± 5.413^∗^	17.601 ± 2.908^∗^
AUC_inf_ (*μ*g.h/mL)	6.692 ± 1.338	12.45 ± 6.326	34.686 ± 5.635^∗∗∗^
CL (L/h/kg)	0.680 ± 0.136	0.206 ± 0.026^∗∗∗∗^	0.081 ± 0.018^∗∗∗∗^

*C*_0_: initial plasma concentration; *t*_1/2_ *α*: half-life in the *α* phase; *t*_1/2_ *β*: half-life in the *β* phase; AUC_inf_: area under the curve (from 0 to infinity); MRT: mean residence time; CL: clearance; ^∗^statistically significant difference compared with L-OHP group (^∗^*P* < 0.05, ^∗∗∗^*P* < 0.001, and ^∗∗∗∗^*P* < 0.0001).

## Data Availability

The data used to support the findings of this study are included in this published article (and its supplementary materials file).

## Supplementary Materials

Supplementary Materials Supplemental methods: HPLC method validation for the quantification of L-OHP in aqueous media. Supplemental results: HPLC method validation for the quantification of L-OHP. Supplemental Tables: Table S1: precision of the HPLC method; Table S2: recovery of L-OHP by HPLC method. Supplemental figures: Figure S1: chromatograms of L-OHP solution (A), 10% TritonX-100 solution (B), and blank liposomes (C); Figure S2: HPLC calibration curve of L-OHP; Figure S3: chromatogram of L-OHP limit of quantification (LOQ); Figure S4: calibration curve of platinum standard in rat plasma; Figure S5: the absorbance of platinum in blank rat plasma (A) and the absorbance of platinum in rat plasma spiked with platinum at LOQ level (B); Figure S6: platinum absorbance of blank rat plasma (A) and specific platinum absorbance of rat plasma at 15 min after L-OHP intravenous injection (B).
