# Supplementary material for: Characterization and Pharmacokinetic Evaluation of Oxaliplatin Long-Circulating Liposomes
Source: Biomed Res Int. 2021 Apr 20;2021:5949804. doi: 10.1155/2021/5949804 (PMC8079196; doi:10.1155/2021/5949804)
Supplement: Supplementary Materials — Supplemental methods: HPLC method validation for the quantification of L-OHP in aqueous media. Supplemental results: HPLC method validation for the quantification of L-OHP. Supplemental Tables: Table S1: precision of the HPLC method; Table S2: recovery of L-OHP by HPLC method. Supplemental figures: Figure S1: chromatograms of L-OHP solution (A), 10% TritonX-100 solution (B), and blank liposomes (C); Figure S2: HPLC calibration curve of L-OHP; Figure S3: chromatogram of L-OHP limit of quantification (LOQ); Figure S4: calibration curve of platinum standard in rat plasma; Figure S5: the absorbance of platinum in blank rat plasma (A) and the absorbance of platinum in rat plasma spiked with platinum at LOQ level (B); Figure S6: platinum absorbance of blank rat plasma (A) and specific platinum absorbance of rat plasma at 15 min after L-OHP intravenous injection (B). [file 5949804.f1.docx]

**Characterization and Pharmacokinetic Evaluation of Oxaliplatin Long-Circulating Liposomes**

Nihad Cheraga ^1^, Ammar Ouahab ^2^, Yan Shen ^2^, Ning-Ping Huang ^1^*

^1^State Key Laboratory of Bioelectronics, School of Biological Science and Medical Engineering, Southeast University, Nanjing, 210096, China.

^2^ Department of Pharmaceutics, China Pharmaceutical University, Nanjing 210009, China

* Corresponding author E-mail: nphuang@seu.edu.cn

**Supplementary methods**

**1. HPLC method validation for the quantification of L-OHP**

Quantification of L-OHP in liposomes formulation or aqueous medium can be achieved by HPLC technique. The method was validated according to the international conference of harmonization (ICH) guideline Q2(R1) [1].

**1.1. Apparatus and chromatographic conditions**

High performance liquid chromatographic (HPLC) method was adopted for the determination of L-OHP concentration. The HPLC system was equipped with a LPG-3400SD pump and a VWD-3100 UV detector (Dionex, USA). The data were analyzed using Chromeleon 6.8 SR11 Build 3161 software. The mobile phase was a mixture of water and methanol (95:5, v/v) at 1mL flow rate. The analysis was conducted on intersil ODS C18 column (4.6 mm × 250 mm, 5 μm) with a detection wavelength of 250 nm. The column was maintained at 30ºC throughout the analysis. The injection volume was 20μl and the chromatogram was recorded for 20 min.

**1.2. Specificity**

L-OHP peak should have no interference from other chemicals or excipients employed in the formulation. Assay interference was investigated by injecting L-OHP solution and 10% Triton X-100 aqueous solution to the HPLC. In addition, blank liposomes were also injected after their disruption by the addition of 10% Triton X-100. All the chromatograms were recorded under the HPLC conditions mentioned above.

**1.3. Linearity**

The linearity is the ability within a given range to obtain responses which are directly proportional to the concentration of the drug. Linearity was studied in the concentration range of 5-100 μg/mL. Stock standard solution of L-OHP (1mg/mL) was prepared by dissolving appropriate amount of L-OHP in mobile phase. 5 mL alquilots was then transferred into 50 mL volumetric flask, to prepare a working solution of 100μg/mL. A series of six standard solutions were prepared by subsequent dilution of the working solution with mobile phase and then assayed in triplicates. linearity of the analytical procedure was evaluated by plotting peak area (y) against analyzed concentration (x).

**1.4. Precision**

Inter-day and within-day precision of three samples of L-OHP solutions (5, 50 and 100 μg/mL) were determined five times per day and once per day for five consecutive days. The precision is expressed as the percent relative standard deviation (RSD%).

**1.5. Recovery**

L-OHP solution at three concentration levels of 10, 50, 100 μg/mL was prepared and analyzed by HPLC. The measured concentration was obtained using calibration curve. The recovery was expressed in percentage by comparing the measured concentration to the nominal concentration.

**Supplementary results**

**1. HPLC method validation for the quantification of L-OHP**

**1.1. Specificity**

The results of assay interference are shown in Figure S1. The chromatogram of L-OHP solution showed a specific L-OHP peak having a retention time of 9.2 min. The chromatograms of blank liposomes and 10% Triton-X100 solution showed that there are no peaks interfering from the excipients used in the formulation.

**1.2. Linearity**

The regression equation was y = 0.1216x- 0.0015 with R2 = 0.9997. The correlation coefficient (R2) obtained demonstrate that there was excellent linearity between peak area and concentration of L-OHP. The results are showed in Figure S2.

**1.3. Precision of the analytical method**

The precision of the method is listed in Table S1. The RSD values of L-OHP at three concentration levels were ranged from 0.61-0.67% for the intra-day precision and from 0.92-1.38% for inter-day precision. The RSD values were ≤ 2% suggesting high precision of this method.

**1.4. Recovery of the analytical method**

The results of recoveries are summarized in Table S2. The recoveries of the three nominal concentrations assessed were ranged from 100.19 to 101.04%, which are within [90-110%] range that is considered as acceptable limits for a pharmaceutical matrix assay method determined

by ICH [1].

**1.5. Limits of detection (LOD) and quantification (LOQ)**

The limits of detection (LOD) and quantification (LOQ) and for L-OHP were determined at a signal-to-noise ratio of 3 and 10, respectively, by injecting a sequence of dilute solutions with known concentration and analyzing them using the same chromatographic condition described above. The limits of detection and quantification obtained were calculated as 0.060 𝜇g/mL and 0.200 𝜇g/mL, respectively (Figure S3). Therefore. This developed method is sensible for L-OHP quantification in pharmaceutical formulations and in aqueous medium.

**Supplementary tables**

Table S1. Precision of the HPLC method

| Conc.  (µg/ml) | Intra-day | | Inter-day | |
| --- | --- | --- | --- | --- |
|  | Mean ±SD | RSD% | Mean ± SD | RSD% |
| 5 | 5.05 ± 0.03 | 0.61 | 5.01 ± 0.07 | 1.38 |
| 50 | 49.48 ± 0.36 | 0.73 | 49.23 ± 0.51 | 1.05 |
| 100 | 99.42 ± 0.66 | 0.67 | 100.11 ± 0.92 | 0.92 |

Table S2. Recovery of L-OHP by HPLC method

| Conc.  (µg/mL) | Measured  (µg/mL) | Recovery  (%) | Mean  (%) | RSD  (%) |
| --- | --- | --- | --- | --- |
| 10.016 | 10.14  10.26  9.96 | 101.24  102.43  99.44 | 101.04 | 1.50 |
| 50.032 | 50.44  49.49  50.43 | 100.83  98.92  100.79 | 100.18 | 1.09 |
| 100.024 | 100.64  101.85  100.21 | 100.62  101.83  100.19 | 100.88 | 0.84 |

**Supplementary figures**


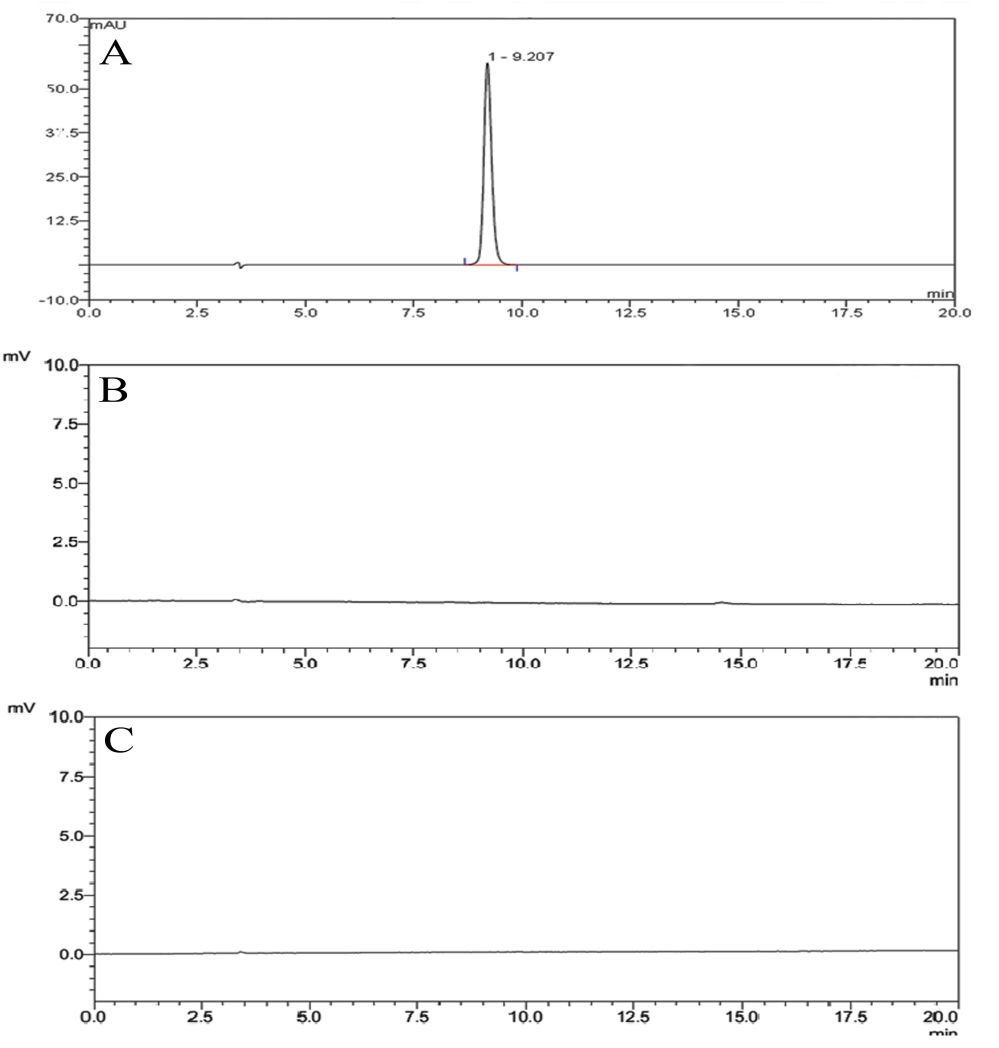


Figure S1. Chromatograms of L-OHP solution (A), 10% TritonX-100 solution (B) and blank liposomes (C).

Figure S2. HPLC calibration curve of L-OHP.


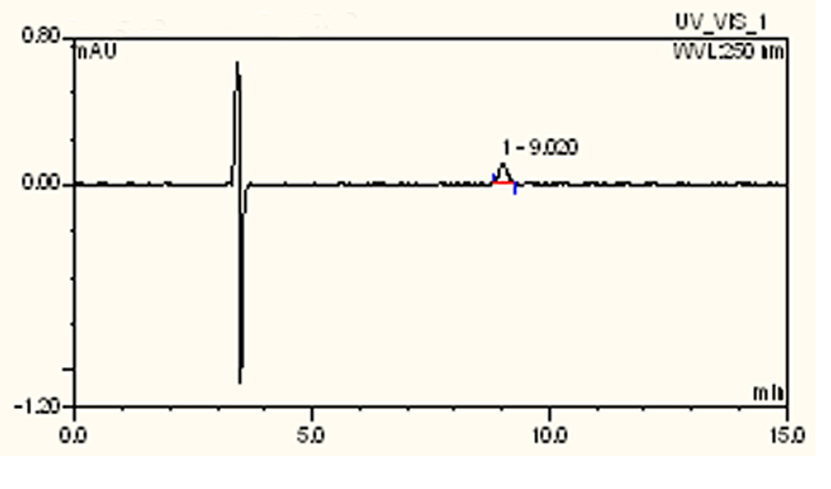


Figure S3. Chromatogram of L-OHP limit of quantification (LOQ).

Figure S4. Calibration curve of platinum standard in rat plasma.

**
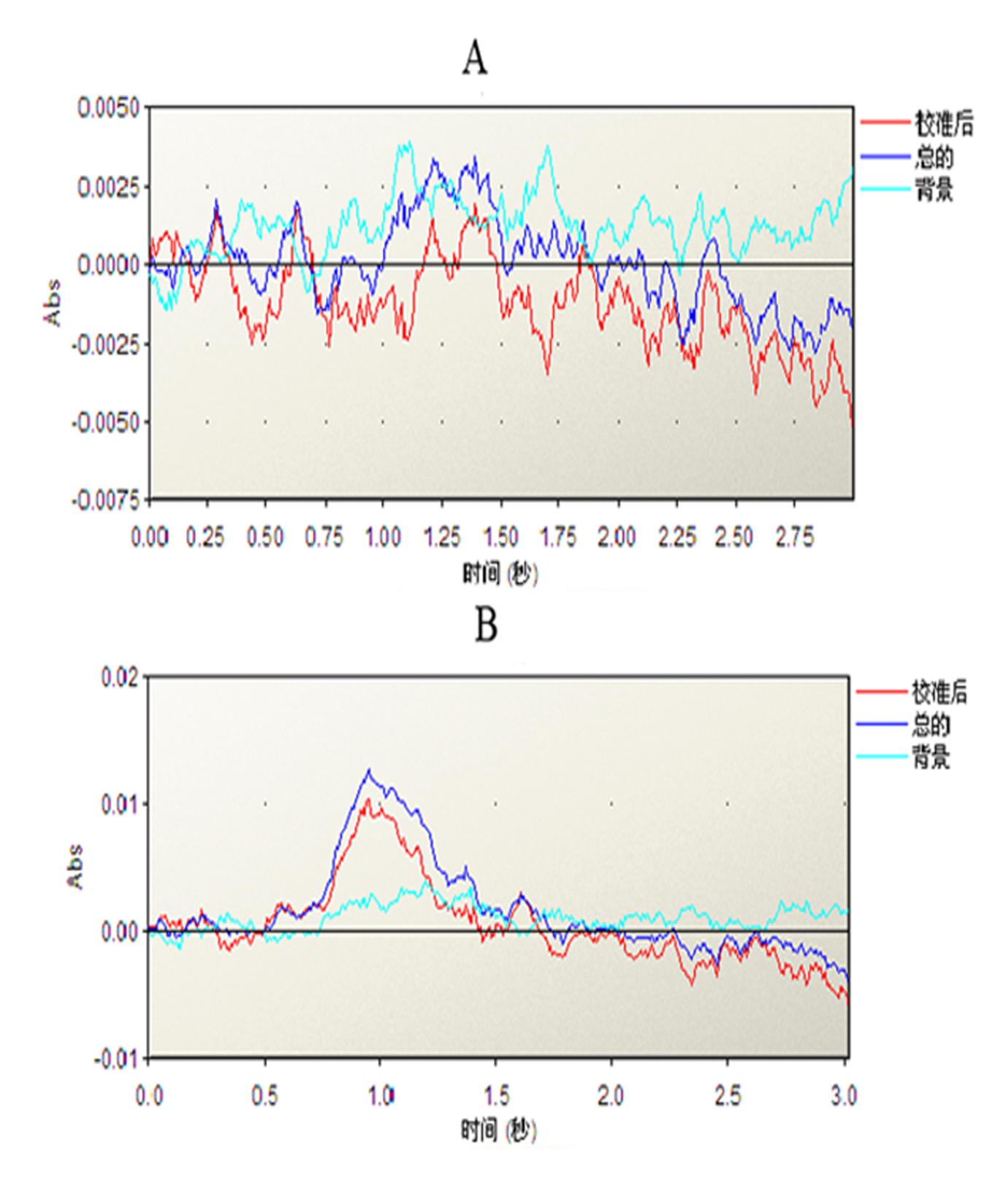
**

Figure S5. The absorbance of platinum in blank rat plasma (A). The absorbance of platinum in rat plasma spiked with platinum at LOQ level (B).

**
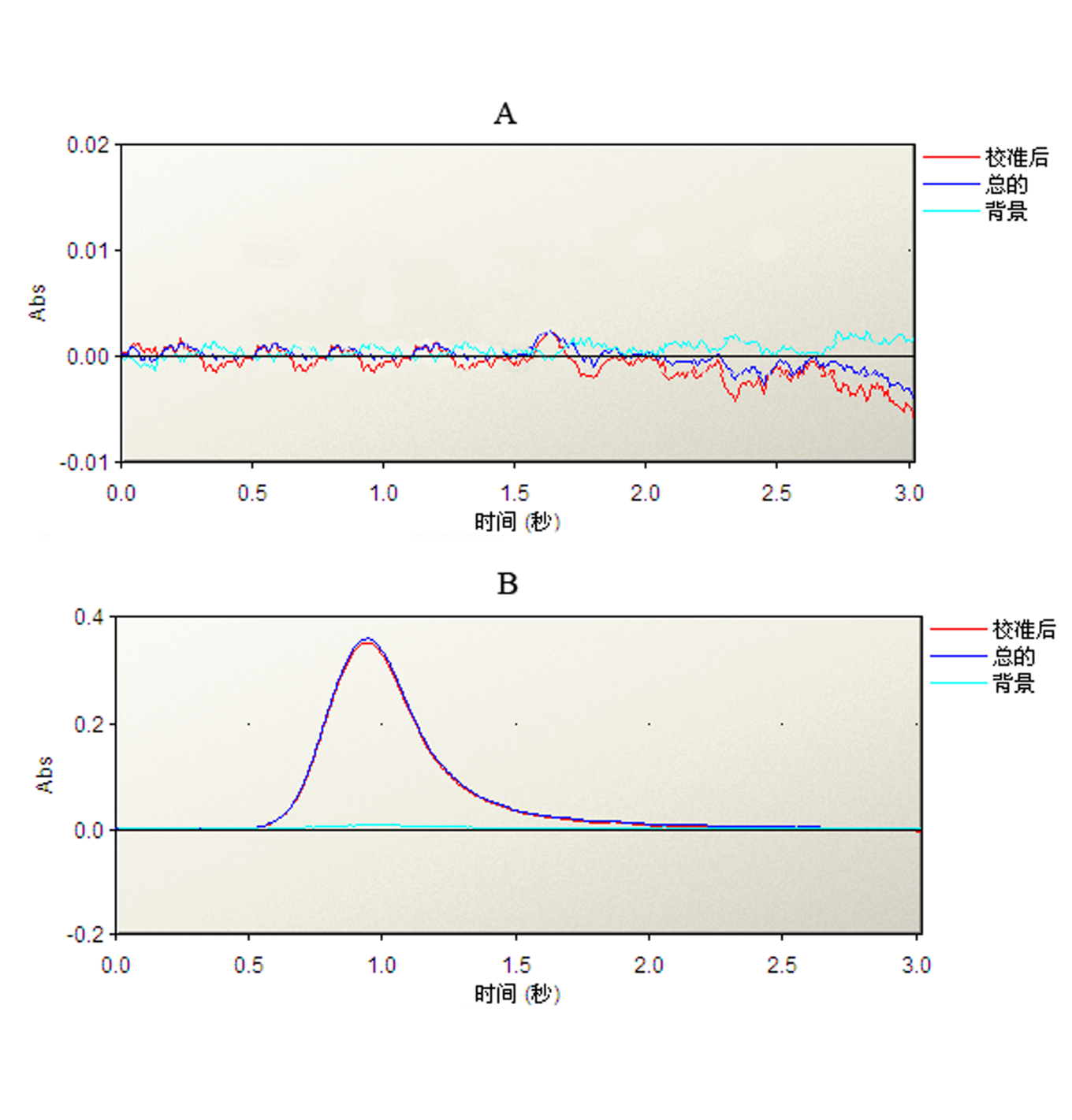
**

Figure S6. Platinum absorbance of blank plasma (A) Specific platinum absorbance of rat plasma at 15 min after L-OHP intravenous injection (B).

References

1. ICH. *Q2(R1), Validation of analytical procedures: text and methodology*. 1995 [cited November 15,2020 ]; Available from: <https://ich.org.ich01.nine.ch/page/quality-guidelines>.
